# Ectopic thyroid gland: clinical features and diagnostics in children

**DOI:** 10.14341/probl12876

**Published:** 2022-02-25

**Authors:** E. V. Shreder, T. A. Vadina, M. B. Konyukhova, E. V. Nagaeva, T. Y. Shiryaeva, S. M. Zakharova, M. V. Degtyarev, E. O. Vyazmenov, O. B. Bezlepkina

**Affiliations:** Endocrinology Research Center;Morozov Children’s Municipal Clinical Hospital; Endocrinology Research Center; Morozov Children’s Municipal Clinical Hospital; Endocrinology Research Center; Endocrinology Research Center; Endocrinology Research Center; Endocrinology Research Center; Endocrinology Research Center; Endocrinology Research Center

**Keywords:** сongenital hypothyroidism, thyroid dysgenesis, ectopia of the thyroid gland, thyroid scintigraphy, ultrasound of the thyroid gland

## Abstract

**BACKGROUND:**

BACKGROUND: The frequency of ectopia of thyroid gland among all types of dysgenesis varies from 30 to 70%, its most common localization is the root of the tongue. Otorhinolaryngologists, oncologists, pediatricians can take lingual ectopia for hypertrophy of the lingual tonsil or fibroma of the tongue root, which leads to unreasonable surgical treatment. Thyroid scintigraphy plays a key role in the diagnosis of ectopia.

**AIM:**

AIM: To assess the etiological structure of congenital hypothyroidism (CH) and demonstrate the clinical course in patients with ectopic thyroid tissue in the root of the tongue.

**MATERIALS AND METHODS:**

MATERIALS AND METHODS: A group of patients with CH was examined. All patients underwent neck ultrasound and radionuclide imaging. The examination was carried out against the background of the abolition of hormone replacement therapy for 14 days or before its initiation. Patients with ectopia in the root of the tongue underwent videofibrolaryngoscopy. Some patients underwent a genetic study with using genes panel of a panel of candidate genes responsible for the development of CH using the NGS method. The molecular genetic study was conducted to some patients, next-generation sequencing with the genes panel.

**RESULTS:**

RESULTS: The study included 73 patients with primary CH aged from 2 weeks to 17.3 years: 69 children were diagnosed based on the results of neonatal screening, 4 children with thyroid ectopia were first examined older than 6 years. The median age of patients at the time of the examination was 6.9 years [4.8; 10.0]. By data of ultrasound aplasia was diagnosed in 47.9% of patients, one child had hemiagenesis and ectopic thyroid tissue of various localization was detected in 26.0% of  children. In 24.7% of children thyroid tissue was found in a typical location. Scintigraphy confirmed thyroid aplasia in 65.7% of children. Examination revealed various variants of ectopically located thyroid tissue in 31 children (42.4%): thyroid ectopia in the root of the tongue in 25 children (80.6%), ectopia in the sublingual region in 5 children (16.2%), double ectopia was detected in 1 child. The median level of TSH in newborns with ectopic thyroid gland was 124 IU/ml and was significantly lower than in children with aplasia — 219 IU/ml, p<0.05. On the other side the level of TG in children with ectopia was significantly higher than in children with aplasia — 37.12 ng/ml versus 0.82 ng/ml, p><0.05. CONCLUSION: Combination of two methods is the best diagnostic approach to determine the etiology of CH — ultrasound and scintigraphy studies compensates deficiencies of each other. Our study demonstrates the importance of scintigraphy in children with CH and patients with the formation of the root of the tongue and the anterior surface of the neck in order to avoid unnecessary removal of the thyroid gland. In case of confirmation of thyroid ectopia in the root of the tongue and in the absence of symptoms of obstruction or bleeding, it is recommended to refer the patient to an endocrinologist for conservative treatment. ><0.05. On the other side the level of TG in children with ectopia was significantly higher than in children with aplasia — 37.12 ng/ml versus 0.82 ng/ml, p< 0.05.

**CONCLUSION:**

CONCLUSION: Combination of two methods is the best diagnostic approach to determine the etiology of CH — ultrasound and scintigraphy studies compensates deficiencies of each other. Our study demonstrates the importance of scintigraphy in children with CH and patients with the formation of the root of the tongue and the anterior surface of the neck in order to avoid unnecessary removal of the thyroid gland. In case of confirmation of thyroid ectopia in the root of the tongue and in the absence of symptoms of obstruction or bleeding, it is recommended to refer the patient to an endocrinologist for conservative treatment.

## Abstract (ru)

**ОБОСНОВАНИЕ:**

ОБОСНОВАНИЕ. Частота эктопии среди всех вариантов дисгенезии варьирует от 30 до 70%, наиболее частая ее локализация — корень языка. Оториноларингологами, онкологами, педиатрами язычная эктопия может приниматься за гипертрофию язычной миндалины или фиброму корня языка, что приводит к необоснованному хирургическому лечению. Ключевую роль в диагностике эктопии играет сцинтиграфия щитовидной железы (ЩЖ).

**ЦЕЛЬ:**

ЦЕЛЬ. Оценить этиологическую структуру врожденного гипотиреоза (ВГ) и продемонстрировать клиническое течение у пациентов с эктопией тиреоидной ткани в корень языка.

**МАТЕРИАЛЫ И МЕТОДЫ:**

МАТЕРИАЛЫ И МЕТОДЫ. Обследована группа пациентов с ВГ. Всем пациентам проведены УЗИ шеи, радионуклидные исследования. Обследования проводились на фоне отмены гормональной терапии в течение 14 дней или до ее начала. Пациентам с эктопией в корень языка проведена видеофиброларингоскопия. Части пациентов проведено молекулярно-генетическое исследование панели генов-кандидатов, ответственных за развитие ВГ методом NGS.

**РЕЗУЛЬТАТЫ:**

РЕЗУЛЬТАТЫ. В исследование включены 73 пациента с первичным ВГ в возрасте от 2 нед до 17,3 года. Медиана возраста пациентов на момент обследования составляла 6,9 года [4,8; 10,0]. 69 детям диагноз установлен по результатам неонатального скрининга, 4 детям с эктопией ЩЖ — после 6 лет. При проведении УЗИ аплазия диагностирована у 47,9% пациентов, у одного — гемиагенезия и у 26,0% — эктопия ткани ЩЖ различной локализации. У 24,7% детей тиреоидная ткань обнаружена в типичном месте. При проведении сцинтиграфии аплазия ЩЖ подтверждена у 65,7% детей. У 31 ребенка (42,4%) выявлены различные варианты эктопически расположенной ткани ЩЖ: эктопия ЩЖ в корень языка имелась у 25 детей (80,6%), эктопия в подъязычную область — у 5 детей (16,2%), двойная эктопия выявлена у 1 ребенка. Уровень неонатального тиреотропного гормона у детей с эктопией ЩЖ составил 124 МЕ/мл и был достоверно ниже, чем у детей с аплазией, — 219 МЕ/мл, р<0,05. Напротив, уровень тиреоглобулина у детей с эктопией был достоверно выше, чем при аплазии, — 37,12 нг/мл против 0,82 нг/мл, р><0,05. ЗАКЛЮЧЕНИЕ. В диагностике этиологии ВГ оптимальным является сочетание двух методов — УЗИ и тиреосцинтиграфии, поскольку каждый метод компенсирует недостатки другого. Проведенное нами исследование демонстрирует важность проведения сцинтиграфии детям с ВГ и пациентам с образованием корня языка и передней поверхности шеи. При подтверждении эктопии ЩЖ в корень языка и при отсутствии симптомов обструкции или кровотечения рекомендовано направить пациента к эндокринологу для назначения консервативного лечения. >< 0,05. Напротив, уровень тиреоглобулина у детей с эктопией был достоверно выше, чем при аплазии, — 37,12 нг/мл против 0,82 нг/мл, р<0,05. ЗАКЛЮЧЕНИЕ. В диагностике этиологии ВГ оптимальным является сочетание двух методов — УЗИ и тиреосцинтиграфии, поскольку каждый метод компенсирует недостатки другого. Проведенное нами исследование демонстрирует важность проведения сцинтиграфии детям с ВГ и пациентам с образованием корня языка и передней поверхности шеи. При подтверждении эктопии ЩЖ в корень языка и при отсутствии симптомов обструкции или кровотечения рекомендовано направить пациента к эндокринологу для назначения консервативного лечения. >< 0,05.

## RELEVANCE

Congenital hypothyroidism (CH) is a frequent congenital disorder of the thyroid gland in children. In Russia, CH incidence is one per 3,617 newborns (one per 2,379 to one per 4,752 in various federal districts) [1]. Most often, CH is caused by thyroid dysgenesis; according to various studies, such dysgenesis accounts for 70%–85% of CH cases [2–8]. Thyroid dysgenesis is subdivided into aplasia, ectopy, hypoplasia, and haemiagenesis.

The incidence of ectopy within all types of thyroid dysgenesis varies from 30% to 70%. Most often such ectopy occurs in the root of tongue [4][6][8–12]. According to autopsy data, the incidence of lingual ectopy is 9.8% [13]. Within ectopic tissue, nodular growths may appear and Hashimoto’s disease may develop [11]; the incidence of thyroid carcinoma and root of tongue carcinoma is very low: the literature reports only isolated clinical cases [14–22].

The first case of lingual ectopy was reported by W. Hickman in 1869. It was a newborn female who died of suffocation 16 hours after birth [23].

Thyroid ectopy may remain undiagnosed for long periods; more often than not this disease is diagnosed before the age of 18 [24]. At the time of diagnosis, thyroid function may be either intact or reduced [25–27]. In rare cases, such symptoms as dysphagia, foreign body sensation, cough, voice distortion, snoring, and sleep apnoea may occur; in more severe cases, there may be haemorrhage and respiratory obstruction [28–35]. Acuteness of clinical presentation will depend on ectopic thyroid tissue size and localisation.

Otorhinolaryngologists, oncologists, and paediatricians can take lingual ectopy for hypertrophy of the lingual tonsil or fibroma of the tongue root, thus initiating surgical intervention.

Thyroid scintiscan plays a key role in the diagnostics of ectopy. In present-day Russia, children with CH normally undergo a thyroid ultrasound only; however, this scan is unable to detect most variants of this ectopy.

## OBJECTIVE

Analyse the etiological structure of congenital hypothyroidism (CH) and demonstrate the clinical course in patients with ectopic thyroid tissue in the root of tongue.

## MATERIALS AND METHODS

## Venue and study start and end dates

Venue. The patients were examined at Children’s Endocrinology Institute, National Endocrinology Research Centre (Moscow).

Start and end dates: Thus study included patients examined between November 2020 and October 2021.

## Observed population(s)

Inclusion criteria: children aged 0–18 with CH where the patient or their parents/legal guardians provided an informed consent to participate in the study.

Exclusion criteria: withdrawal of the patient’s or their legal guardians’ consent to participate in the study.

Population sampling method: unselected sampling.

## Research design

A single-centre, interventional, cross sectional, non-comparative study which included 73 patients with primary CH aged from 2 weeks to 17.3 years. Patients were included in the groups based on inclusion criteria, subject to exclusion criteria. All patients underwent neck ultrasound and radionuclide imaging: a sweep scan and a single-photon emission CT (SPEСT) with 99mТc pertechnetate. Thyroglobulin measurement and scintigraphy were performed 14 days after hormone replacement therapy suspension or prior to its initiation. Patients with ectopy in the root of tongue were attended by an otorhinolaryngologist and underwent videofibrolaryngoscopy.

## Medical intervention (for interventional studies)

Laboratory tests: serum thyroid stimulating hormone (TSH), free T4, free T3, and thyroglobulin measurements were performed at National Endocrinology Research Centre Laboratory (Director: Ms. L. V. Nikankina). The ARCHITECT i2000sr immunochemiluminiscent analyser (Abbott) was used to run the lab tests.

Ultrasound scans were performed by ultrasound imaging physician Ms. S. M. Zakharova at National Endocrinology Research Centre Consulting & Diagnostics Facility (Director: Professor N. N. Volevodz). Voluson E8 expert (GE Healthcare) with 11L linear transducer was operated by an expert level assistant. Colour Doppler imaging and power Doppler imaging was used for the scans.

Genetic tests were conducted with three children having lingual ectopy; these patients are described in this article in detail. The tests were performed at the Laboratory of Monogenic Endocrine Diseases Institute, National Endocrinology Research Centre (Director: Mr. P. Yu. Volchkov). Blood samples were taken from cubital veins irrespective of meal timing. Vials with conserving agent, ethylene diaminetetraacetic acid (EDTA) (1.2 to 2.0 mg per 1 ml of blood) were used for the samples. Test method: next-generation sequencing (NGS) with paired-end tags (150×2) on the Illumiuna platform. Average depth of coverage: 111×; breadth of coverage (10×): 98%. CH Primer panel covers coding regions of 23 genes: AITD3, DUOX1, DUOX2, DOUXA2, FOXE1, GLIS3, GNAS, IYD, NKX2-1, NKX2-5, PAX8, SECISBP2, SLC16A2, SLC26A4, SLC5A5, THRA, THRB, TPO, TRH, TRHR, TSHB, TSHR, and UBR1.

Thyroid scintiscans (in the neck and upper mediastinal regions) were performed at Radionuclide Diagnostics Department, National Endocrinology Research Centre (Director: Mr. M. V. Degtyarev) with a Discovery NM630 SPECT gamma camera with99mТc pertechnetate. The radiopharmaceutical dose was determined individually based on the patient’s weight with PedDose calculator in MBq and mCi (https://www.eanm.org/publications/dosage-calculator). Sodium 99mТc pertechnetate solution was obtained by99Мо/99mTc generator elution with a sterile isotonic solution of sodium chloride. The radiopharmaceutical thus obtained from the generator was administered intravenously in a treatment room. Tests were conducted 15 to 20 minutes after radiopharmaceutical administration with a gamma camera; static planar images were taken while patients remained in dorsal position (10 minutes) and then SPECT images were taken (15 minutes). Data processing was performed with Xeleris workstation (GE Healthcare); iterative data reconstruction was applied resulting in three-dimensional images of radiopharmaceutical distribution across the tissues in the neck and upper mediastinal regions. Anatomic and physiological properties of visualised thyroid tissue were then described.

Videofibrolaryngoscopy was performed by an otorhinolaryngologist Mr. E. O. Vyazmenov at National Endocrinology Research Centre Consulting & Diagnostics Facility (Director: Professor N. N. Volevodz) with a Pentax machine.

## Statistical analysis

No prior calculation of sample size was carried out. RStudio (Version 1.1.463 — © 2009–2018 RStudio, Inc.) with R Suite (Version 3.5.3) was used for statistical processing. Shapiro-Wilk’s test was applied to verify distribution normality. Quantitative data were presented as a median and interquartile range (Me [Q1; Q3]).

## Ethical review

Research protocol was approved by Endocrinology Research Centre’s internal Ethics Committee on 28 October 2020 (official session record no. 17). Written informed consent was provided by each participant, or their guardian or legal representative.

## FINDINGS

73 patients with primary CH aged from 2 weeks to 17.3 years were included in the study. The patients’ median age at the time of examination was 6.9 years [ 4.8; 10.0]. Sex distribution within the sample: 48 females and 25 males. 69 children were diagnosed basedon the results of neonatal screening; three children with thyroid ectopy were diagnosed after the age of 6, and one child with a sublingual ectopy was diagnosed at the age of 15.8. Throughout the study, none of the patients was excluded.

In none of these cases ectopic tissue been detected through ultrasound scans made at GP clinics. Colour Doppler imaging performed at National Endocrinology Research Centre revealed aplasia in 47.9% of patients; ectopic thyroid tissue of various localization was detected in 26.0% of children; one case of hemiagenesis was diagnosed. In 24.7% of children thyroid tissue was found in a regular location. This group included children with hypoplasia (n=9) and those with goitre (n=9) (Figure 1, a). Median volume of thyroid gland inchildren with hypoplasia was 1.2 ml [ 0.2; 1.9]; in children with goitre, it amounted to 7 ml [ 5.3; 8.9].

Thyroid scintiscans confirmed thyroid aplasia in 65.7% of patients (23 out 35). Thyroid tissue localisation was confirmed by thyroid scintiscan data in all 18 children with gland-in-situ and in one child who was diagnosed with haemiagenesis through ultrasound scan. (Figure 1, b).

**Figure:**
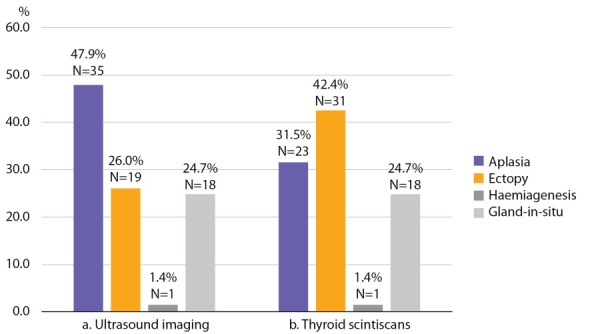
Figure 1. Thyroid tissue visualisation based on ultrasound imaging (a) and thyroid scintiscans (b)

Thus, visualising methods revealed various kinds of thyroid ectopic tissue in 31 patients (42.4%):

According to literature, thyroid ectopy occurs in 48.4% of children with CH and is the most frequent cause of dysgenesis in CH. In our study, CH caused by thyroid ectopy was diagnosed through neonatal screening in the absolute majority of cases (87.1%).

Neonatal TSH level in patients with thyroid ectopy was significantly lower than in those with aplasia. The level of thyroglobulin, on the contrary, was significantly higher in patients with ectopy than in those with aplasia. We have not established any significant differences in levothyroxine average daily dose between patients with aplasia and those with ectopy (see Table 1).

**Table:** Table 1. Neonatal TSH, thyroglobulin and levothyroxine average daily dose in children with CH

Type of thyroid dysgenesis	Number of patients, N	Neonatal TSH (normal level: up to 9 MU/ml)	Thyroglobulin (normal level: 3.5–77 ng/ml)	Levothyroxine average daily dose (mg/kg/day)
Aplasia	23	219 [ 194.0; 343.0]	0.82 [ 0.04; 6.28]	2.9 [ 2.4; 3.4]
Ectopy	31	124 [ 63.0; 252.0]	31.12 [ 23.2; 64.0]	2.6 [ 2.0; 3.0]
p		<0.05	<0.05	>0.05

We are presenting below three clinical cases of ectopy in the root of tongue; these patients were diagnosed with CH after the age of 6; two of the patients underwent removal of ectopic thyroid tissue (their ectopic thyroid tissue had been mistaken for lingual tonsil hypertrophy or for root of tongue fibroma). These cases highlight the importance of running thyroid scintiscans to diagnose thyroid ectopy properly.

## Case 1

An 8-year-old female first was examined about neoplasm at the root of tongue at the age of 6.5. The patient was followed to ENT and diagnosed with lingual tonsil hypertrophy (Figure 2).

**Figure:**
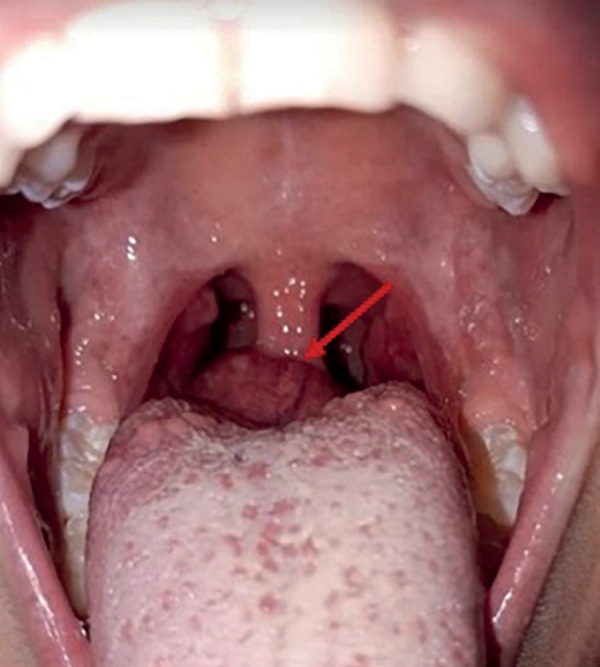
Figure 2. Ectopic thyroid tissue in the root of tongue observed during pharyngoscopy at the age of 6.5

The patient was admitted to a surgical department and diagnosed with root of tongue fibroma. Based on MRI images of her neck region, a “nodular appearance at the root of tongue” was confirmed and surgical removal was performed. Histology found it to be thyroid tissue. 3 weeks after the surgery, the patient was diagnosed with hypothyroidism (TSH — 206 mU/L; free T4 — 5.02 pmol/L). Hormone therapy with levothyroxine was recommended.

At the age of 7.5 the patient came to the National Endocrinology Research Centre for the first time. An ultrasound imaging found residual thyroid tissue at the root of tongue with dimensions 1.0×0.7×0.5 cm. For the first time, a cyst in the left-hand side of anterior neck was identified with dimensions 0.4×0.3×0.2 cm (Figure 3, a). A thyroid scintiscan confirmed the presence of a round-shaped residual ectopic thyroid tissue 1.4×1.2×1.5 cm in the root of tongue (Figure 3, b). The patient has continued to receive hormone therapy with 2.4 mg/kg/day levothyroxine.

**Figure:**
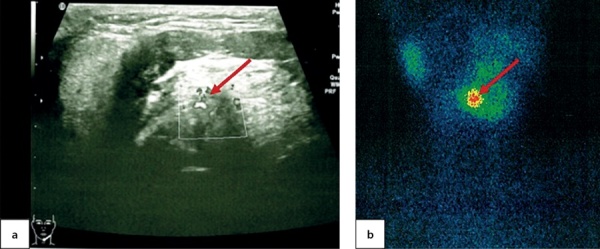


## Case 2

A 6.5-year-old female reported having periodic cough. During examination the growth at the root of tongue was confirmed by CT and MRI imaging (dimensions: 1.4×1.9×1.3 cm). The patient was admitted to an oncology department and advised surgical removal of the growth. Tumour markers were negative (α-fetoprotein 3.11 ng/ml; β-subunit of human chorionic gonadotropin 1.2 mIU/mL). The patient underwent surgical removal of the growth. Histology found it to be thyroid tissue. At a 3-week follow-up examination, the patient was diagnosed with hypothyroidism (TSH — 108 mU/L; free T4 — 7.61 pmol/L). Hormone therapy with levothyroxine was recommended.

At the age of 6.5 the patient came to the National Endocrinology Research Centre for the first time. An ultrasound imaging found evidence of thyroid aplasia and identified a cyst in the right-hand side of anterior neck with dimensions 1.0×0.6×0.7 cm (Figure 4, a). Athyroid scintiscan confirmed the presence of a round-shaped residual ectopic thyroid tissue in the root of tongue and established its dimensions as 1.0×1.2×1.0 cm (Figure 4, b). The patient has continued to receive hormone therapy with 2.5 mg/kg/day levothyroxine.

**Figure:**
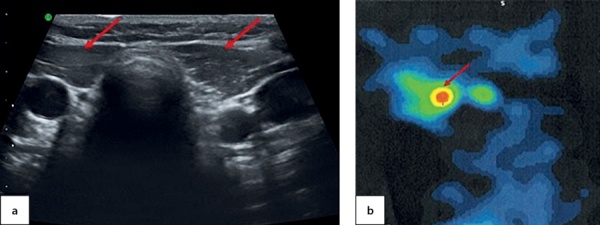


## Case 3

A 6.5-year-old girl experienced discomfort when swallowing. Her parents noticed a growth in the root of tongue region. A CT scan showed a strong accumulation of contrast agent by a growth in the root of tongue region with the size 1.4×1.6×1.7 cm. Ultrasound imaging identified no thyroid gland in situ. The patient’s hormonal profile had evidence of subclinical hypothyroidism (TSH — 5.83 mU/L; free T4 — 16.1 pmol/L). Levothyroxine treatment with 0.6 mg/kg/day was recommended.

At the age of 7.5, this patient the patient was admitted the National Endocrinology Research Centre for the first time. She complained of pain while swallowing and periodic cough. During pharyngoscopy a growth at the root of tongue was observed (Figure 5, a). Thepatient was seen by an otorhinolaryngologist; through a videofibrolaryngoscopy, a nodular growth was clearly identified (Figure 5, b).

**Figure:**
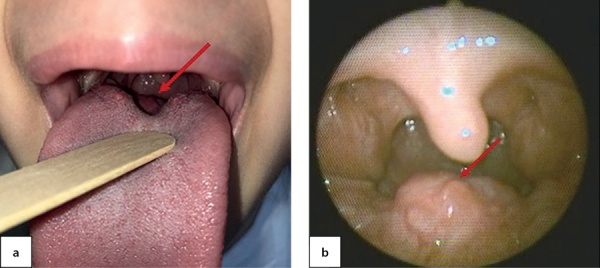
Figure 5. Ectopic thyroid tissue in the root of tongue as observed through pharyngoscopy (a) and videofibrolaryngoscopy (b) in a 7.5-year-old patient

An ultrasound imaging found a round-shaped thyroid tissue in the root of tongue plane; it had low echoicity, homogenous structure and active vascularisation when observed through colour Doppler imaging and power Doppler imaging (Figure 6, a & b). Moreover, acyst was identified in the right-hand side of anterior neck with dimensions 0.4×0.3×0.2 cm. A thyroid scintiscan confirmed the presence of ectopic thyroid tissue in the root of tongue with dimensions 1.4×1.5×1.8 cm (Figure 6, c). The dose of levothyroxine for this patient was increased to 1.3 mg/kg/day.

**Figure:**
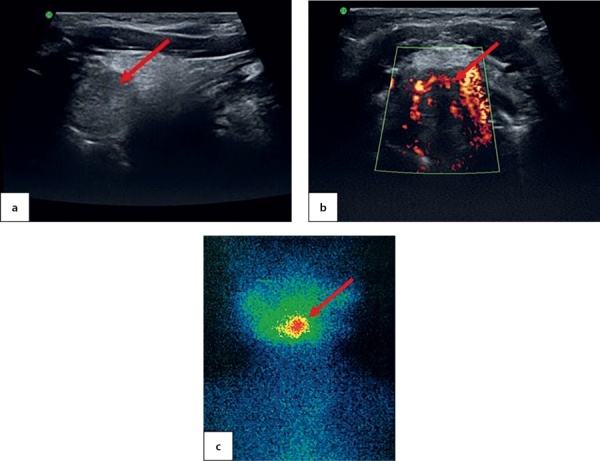


Since thyroid dysgenesis is most often caused by mutations in the genes PAX8, NKX2-1, NKX2-5, TSHR, and FOXE1 [36], these three patients underwent a genetic test. No clinically significant changes were found in any of these cases.

## DISCUSSION

Based on comprehensive laboratory and instrumental evaluation, our study found that 87.7% of CH patients (64 out of 73) had thyroid dysgenesis, which correlates with international data [28]. Within our group of patients, thyroid dysgenesis is subdivided into ectopy (48.4%), aplasia (35.9%), hypoplasia (14.1%) and thyroid haemiagenesis (1 patient). In international studies, ectopy is likewise the most widespread condition in CH patients (40–60%) [3][8][37–41]. Aplasia accounts for 15–30% of cases, and hypoplasia for about 5% ofcases [9][37–38][42].

In our study, the function of ectopic thyroid tissue varied from subclinical to manifest hypothyroidism. In 94.5% of patients, CH was diagnosed through neonatal screening; in four patients, ectopy was identified after the age of 6. Two patients underwent surgical removal of ectopic thyroid tissue.

The three patients we have described had different clinical manifestations. In the first of these cases, ectopy was identified by a paediatrician during an examination after recovery from tonsillitis. In the second one, periodic cough was the only clinical manifestation.In the third one, a sizeable growth manifested through dysphagia and cough. Similar clinical manifestations caused by ectopic thyroid tissue have been reported in literature and shown to depend on the size of ectopic tissue [28–34][43]. The remaining 22 patients with ectopic thyroid tissue in the root of tongue were diagnosed through neonatal screening, whereas no clinical manifestations (such as cough or dysphagia) were observed. This can be explained by small size of ectopic tissue due to levothyroxine therapy.

In our study, most patients with thyroid ectopy had high neonatal TSH levels; however, these levels were significantly lower than in those with aplasia. Most researchers likewise report high levels of neonatal TSH in children with CH caused by thyroid ectopy. R. Perry et al. found average TSH level in children with ectopy to be 144 mIU/L [4]. E. Karakoc-Aydiner et al. showed that TSH levels in patients with ectopy were above 100 mIU/L (130 mIU/L on average), whereas thyroglobulin level was 34.5 ng/mL [40], which also correlates with our findings.

Our study showed that ultrasound imaging is able to identify ectopy in one patient out of four (26%). Thyroid scintiscans confirmed every ectopy case identified through ultrasound imaging and identified a further 12 cases which had been thitherto mistaken for aplasia. Low specificity of ultrasound imaging has been demonstrated by several studies [44][45]. Ultrasound imaging is a non-invasive and cost-efficient method; however, it lacks in sensitivity and specificity and is thus unable to detect most of ectopy types or evaluate the function of thyroid tissue if one exists. If thyroid tissue is not present in its regular location, regions of its potential ectopy need to be examined.

As we know, cells of human thyroid gland have a double origin. The medial anlage grows out of the medial bulge of the ventral wall of pharynx between the 1st and the 2nd pair of pharyngeal recesses, whereas the two lateral anlage (ultimobranchial bodies) are aproduct of the 4th pair of pharyngeal recesses and the neural crest. In foetus, thyroid gland anlage appears on the 16th or 17th day of foetal development as a cluster of entodermal cells near the root of tongue. Then, this group of cells ingrows into the underlying mesenchymal tissue alongside the pharyngeal intestine up to the level of the 3rd and 4th pairs of pharyngeal pouches and then migrated into the neck area ventral of the laryngeal cartilages. By the end of week 4, thyroid gland anlage takes the shape of a protruding cavity (epithelial bundle) connected with the pharynx through a small opening at the root of tongue, thyrolingual duct. Then the anlage descends to the final location of thyroid gland and pulls the thyrolingual duct with it; the distal end of the bundle bifurcates and thyroid gland lobes develop with an isthmus connecting them. In normal cases, the proximal end of thyrolingual duct atrophies and completely disappears by week 8 of foetal development, leaving behind a vestige [46][47]. Thus, ectopic thyroid tissue can be identified throughout the anlage migration route.

Ultrasound imaging enables one to evaluate thyroid gland structure and dimensions and identify its development anomalies such as thyroglossal duct cysts, thymus cysts or thymus tissue [48]. In this study, we did not evaluate the incidence of cyst growths; however, inall three thyroid ectopy cases cysts on the anterior neck were visualised. It has been suggested that such congenital cysts result from preservation of ultimobranchial bodies or thyrolingual duct during embryogenesis [46].

The key disadvantage of ultrasound imaging in topical diagnostics is its lack of ability to identify thyroid ectopy. This ability grows if colour Doppler imaging is used [40][49]. This disadvantage of ultrasound imaging is compensated by scintiscans.

In Russia, children with CH rarely undergo thyroid scintiscan; most often, this takes place in more senior ages. According to international guidelines [50][51], thyroid scintiscans can be performed for any CH children regardless of age, including newborns [44][52]. This method enables to evaluate CH aetiology and the activity of thyroid tissue if one exists. Typically, sodium 99mТc pertechnetate or iodine-123 (123I) is used as radiopharmaceutical. When performing a thyroid scintiscan in the planar mode, sensitivity amounts to 63%–88% and specificity amounts to 92%–96%. The SPECT mode increases this method’s sensitivity [50][51][53].

The most informative method combines ultrasound imaging with a radiopharmaceutical method of thyroid examination (a planar-mode SPECT scintiscan of the neck and upper mediastinal region). Such double visualisation is especially helpful for aplasia confirmation and thyroid ectopy identification [54][55].

When examining CH patients, methods providing visualisation enable practitioners to make a topical diagnosis and in some cases to distinguish between transitory and permanent forms of the disease; they also enable to set the initial dose of levothyroxine and raisethe parents’ commitment to therapy. Identification of patients with thyroid aplasia or ectopy prior to the start of therapy enables to avoid suspending it in higher ages for diagnostic purposes [56].

If thyroid ectopy is identified relatively late, the treatment strategy will depend on several factors: severity of symptoms, growth size, and thyroid gland function. Patients with euthyroidism and without symptoms of obstruction must be followed up regularly. For those diagnosed with hypothyroidism, levothyroxine should be prescribed in order to reduce TSH and thus remove the factor stimulating thyroid gland growth. Several authors have demonstrated efficient treatment of patients with ectopic thyroid tissue whereby this tissue decreases in size in 60% of patients. Treatment dose and duration should be set individually [24].

Furthermore, it is advisable to measure thyroglobulin level as it is an additional marker of thyroid tissue; once measured, this level should be evaluated together with visualisation results.

## Further research

To follow this study, we are planning to expand the sample size and conduct genetic tests with candidate genes accounting for CH development. The next step will consist in establishing a correlation between clinical variants, genetic data and anatomic/functional visualisation of thyroid tissue.

## CONCLUSION

Thus, ultrasound imaging of thyroid gland enables one to assess in detail its location, size, echoicity, and vascularisation; however, it does not always enable to identify ectopic thyroid tissue. Sensitivity of ultrasound imaging will increase if the scan is made by an experienced operator and with an expert level machine, if all potential locations of ectopic thyroid tissue are examined and colour Doppler imaging is used. On the other hand, thyroid scintiscan does not always enable to determine the size of thyroid tissue with precision; however, it virtually always identifies ectopic thyroid tissue. Therefore, a combination of these two methods is the best diagnostic approach.

Thyroid scintiscan should be conducted as early as possible to arrive at a topical diagnosis.

Our study demonstrates the importance of performing scintiscans when examining children with CH and patients with growths in the root of tongue and the anterior surface of the neck in order to avoid unwarranted surgical removal of thyroid gland. If thyroid ectopy in the root of tongue is confirmed and no symptoms of obstruction or bleeding are present, we recommend to refer the patient to an endocrinologist for conservative treatment.

Since lingual ectopy is the most frequent variant and, as our observations demonstrate, it can be misinterpreted, a timely topical diagnosis is crucial; an equally important task is to raise the awareness of doctors in other fields (paediatricians, otorhinolaryngologists, paediatric surgeons, and oncologists) about this problem.

## ADDITIONAL INFORMATION

Funding source. This study was carried out as part of a Government contract for a protocol clinical trial entitled “A Method of Hybrid Anatomic/Functional Visualisation of Thyroid Tissue for Topical and Functional CH Diagnostics in Children” (no. 2019-15-21) and was co-funded (in the part involving genetic tests) by CAF Foundation as part of Alpha Endo national charity programme.

Conflict of interest. The authors hereby declare no actual or potential conflict of interest related to this publication.

Authors’ contribution. Ekaterina V. Shreder: research concept and design, provision of examination materials, data analysis, article drafting; Olga B. Bezlepkina: research concept and design, article editing, valuable comments; Tatiana A. Vadina & Marina B.  Konyukhova: provision of examination materials; Elena V. Nagaeva & Tatiana Y. Shiryaeva: research concept and design, valuable comments; Svetlana M. Zakharova & Mikhail V. Degtyarev: provision of examination materials, valuable comments; Eduard O. Vyazmenov: provision of examination materials, article editing, valuable comments. Every author approved the final version of the text prior to publication and agreed to accept responsibility for all aspects of this study, which implies due investigation and resolution of any issue related to the accuracy or integrity of any part thereof.

Acknowledgments. The authors express their gratitude to CAF Foundation and Alpha Endo national charity programme for funding the part of this study involving genetic tests.
